# Characterization of acoustic streaming in water and aluminum melt during ultrasonic irradiation

**DOI:** 10.1016/j.ultsonch.2020.105381

**Published:** 2020-10-28

**Authors:** Takuya Yamamoto, Kazuki Kubo, Sergey V. Komarov

**Affiliations:** a Department of Metallurgy, Tohoku University, Miyagi 980-8579, Japan; b Graduate School of Environmental Studies, Tohoku University, Miyagi 980-8579, Japan

**Keywords:** Acoustic streaming, Acoustic cavitation, Vortex shedding frequency, Aluminum melt, Ultrasound irradiation, Karman vortex

## Abstract

It is well known that ultrasonic cavitation causes a steady flow termed acoustic streaming. In the present study, the velocity of acoustic streaming in water and molten aluminum is measured. The method is based on the measurement of oscillation frequency of Karman vortices around a cylinder immersed into liquid. For the case of acoustic streaming in molten metal, such measurements were performed for the first time. Four types of experiments were conducted in the present study: (1) Particle Image Velocimetry (PIV) measurement in a water bath to measure the acoustic streaming velocity visually, (2) frequency measurement of Karman vortices generated around a cylinder in water, and (3) in aluminum melt, and (4) cavitation intensity measurements in molten aluminum. Based on the measurement results (1) and (2), the Strouhal number for acoustic streaming was determined. Then, using the same Strouhal number and measuring oscillation frequency of Karman vortices in aluminum melt, the acoustic streaming velocity was measured. The velocity of acoustic streaming was found to be independent of amplitude of sonotrode tip oscillation both in water and aluminum melt. This can be explained by the effect of acoustic shielding and liquid density.

## Highlights

• Acoustic streaming velocity in aluminum melt and water was measured through Karman vortex frequency measurement.
• The velocity of acoustic streaming is independent of amplitude of horn tip oscillation both in water and aluminum melt.
• The velocity of acoustic streaming in aluminum melt is approximately the same as that in water.

## 1 Introduction

The ultrasonic treatment of molten metals has been proposed in the past as a promising and useful technology for the melt degassing and improvement of the metal solidification structure. Ultrasonic vibrations can be introduced in molten metal through a sonotrode immersed into the melt. In this case several phenomena are occurring simultaneously. First, ultrasound irradiation causes pressure oscillations in melt that can result in acoustic cavitation, and when the cavitation bubbles collapse, high temperature zone (hot spot) and shock waves are generated. Second, the attenuation of ultrasonic energy due to cavitation induces a steady-state flow in melt called acoustic streaming. This flow affects such phenomena as heat and mass transfer that can be responsible for altering the structure and properties of solidified metal. Therefore, understanding and controlling the above phenomena are of critical importance in optimizing conditions of the ultrasonic treatment. However, there is a very limited number of measurement techniques to investigate the acoustic cavitation and acoustic streaming in molten metals because of high temperature and opaqueness. That is the main reason why acoustic cavitation and streaming related phenomena in aluminum melt have not been clarified yet. There exists a very limited amount of literature on experimental and theoretical investigations of these phenomena in molten aluminum.

The cavitation intensity and sound pressure profile in aluminum melt were investigated using high-temperature cavitometers [1], [2], [3], [4], [5]. The cavitometer exploits the principle of spectral analysis of the acoustic emission signal coming from cavitation zone. Besides, a combination of numerical simulation and cavitometer measurement [6] or analytical solution [7] have been proposed to investigate the cavitation-related phenomena. For example, Komarov *et al*. (2013) used a cavitometer to determine the threshold vibration amplitudes of cavitation onset [1]. The results showed that this amplitude in aluminum melt is approximately twice than that in water. The similar results were obtained in Tzanakis *et al*. [3], [4], [5]. Tzanakis *et al*. [3], [4], [5] found that acoustic shielding is stronger in aluminum melt compared with that in water because the sound pressure attenuation is larger in molten aluminum. Lebon *et al*. (2018) also investigated the pressure distribution using a high-temperature cavitometer [6]. They could measure the two-dimensional distribution of sound pressure. Later, the attenuation of ultrasound propagation was analytically investigated by Yamamoto and Komarov (2019) [7]. They also showed that the sound attenuation becomes larger with increasing the size of cavitation bubbles.

In addition to the sound pressure distribution and cavitation intensity, the behavior of cavitation bubbles and their size were investigated by X-ray imaging [8], [9], [10]. Tzanakis *et al*. (2015) investigated a sonocapillary effect in Al-Cu alloy melt [8]. They found that this effect causes the melt to fill a gas pocket of an oxide film and that the implosion of cavitation bubbles is the key factor causing the sonocapillary effect. Xu *et al*. (2016) investigated the size of cavitation bubbles and the time variation of bubbles in size in Al-Cu alloy melt [9]. They found that the bubble size increases with time after stopping the ultrasound irradiation and the most frequently detected averaged radius of bubbles is approximately 15 μm, which is slightly larger compared with that in water [11]. Wang *et al*. (2020) could observe a bubble cloud under a sonotrode using X-ray imaging [10]. The cavitation bubble behavior and characteristics has been revealed in recent years.

On the contrary to the cavitation bubble and ultrasound pressure distribution, characteristics of acoustic streaming in aluminum melt have been investigated in much lesser extent. A proper understanding of acoustic streaming features is important for casting technology including Direct Chill (DC) casting process [12], [13], [14]. Because of the above mentioned experimental difficulties in the case of molten metals, numerical simulation with combination with water model experiments remains the main investigation approach in this area. Fang *et al*. (2018) developed a numerical model to predict the acoustic streaming, cavitation bubble profile and sound pressure distribution [15]. It was found that the shape of sonotrode tip has a significant influence on both the cavitation zone dimension and acoustic streaming pattern. Yamamoto and Komarov (2019) simulated the acoustic streaming in aluminum melt numerically [7]. They found that the velocity of acoustic streaming in aluminum melt is slower than that in water. Based on a different approach, Lebon *et al*. (2019a) developed a numerical model to simulate the acoustic streaming in molten aluminum [16]. They showed that the streaming develops towards the sonotrode tip in the case of low ultrasound power input. Lee *et al*. (2019) investigated the effect of ultrasonic amplitude and frequency on the acoustic streaming [17]. They measured and evaluated the flow velocity of acoustic streaming in water by Particle Image Velocimetry (PIV) measurement and numerical simulation and showed that the velocity of acoustic streaming is inversely proportional to the liquid density. Tzanakis *et al*. (2018) also investigated the acoustic streaming in water by PIV measurement and measured the cavitation intensity [18], and indicated that the direction of acoustic streaming is dependent on the input power of ultrasound.

The all above-mentioned works have aimed at predicting the acoustic streaming in molten aluminum numerically or experimentally using a water model. However, up to now no attempt has been made to measure the acoustic streaming in aluminum melt experimentally.

Therefore, in this study we made an attempt to measure the velocity of acoustic streaming in aluminum melt experimentally based on the Karman vortex frequency measurements behind a cylinder. It is well known that the frequency of Karman vortices depends on the cylinder diameter and fluid flow velocity. Hence, the frequency can be evaluated through a non-dimensional ratio named Strouhal number.

## 2 Experimental method

In the present study, the acoustic streaming velocity is determined from oscillation frequency of a cylinder immersed in the flow [19], [20]. The cylinder is forced to oscillate in the flow by Karman vortices. The frequency of Karman vortices is determined by a non-dimensional Strouhal (*St*) number, which is expressed as:

(1) $$\mathrm{St}=\frac{\mathrm{fD}}{U}$$

where, *f* is frequency of Karman vortices, *D* is the cylinder diameter, and *U* is the flow velocity. Thus, the oscillation frequency depends on the cylinder diameter and flow velocity, and it is independent of the fluid properties when the flow Reynolds number is sufficiently large [19]. Therefore, when the *St* number is known, the fluid velocity can be determined from the oscillation frequency regardless of fluid properties. The Karman vortex measurements is applicable in the range of Reynolds number from 10^2^ to 10^4^, where the flow is transient or turbulent [21]. The Reynolds number is defined as:

(2) Re=UDν

where, ν is the kinematic viscosity. As shown later in detail, the flow velocity is approximately 0.4 m/s, and the cylinder diameters are 0.008–0.012 m. Therefore, the Reynolds number in the present experiment varies from 3000 to 5000 that corresponds to a transient or weakly turbulent flow condition. Therefore, it was assumed that any effect of three-dimensional turbulent flow structures on the acoustic streaming is negligibly small and can be ignored.

**Fig. 1:**
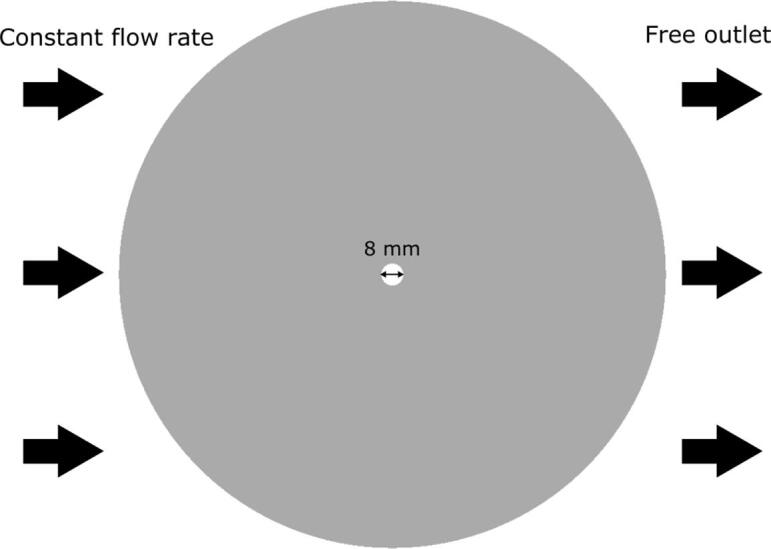
Schematic diagram of two-dimensional calculation domain to simulate the Karman vortex.

**Fig. 2:**
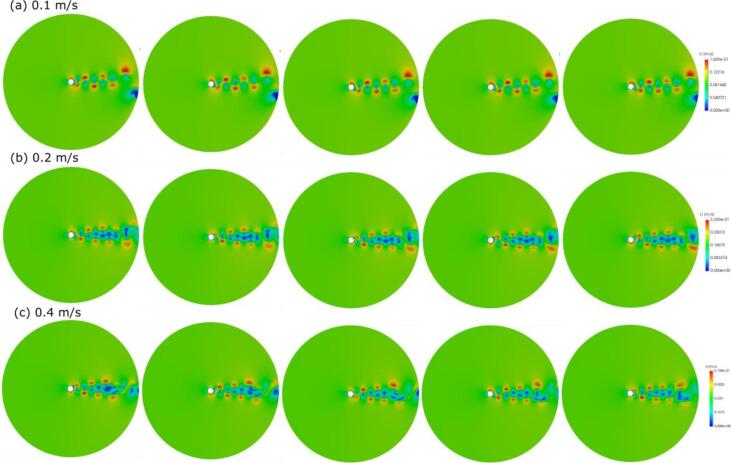
Time variation of velocity contours near the cylinder at different flow velocities: (a) 0.1 m/s, (b) 0.2 m/s, and (c) 0.4 m/s.

**Fig. 3:**
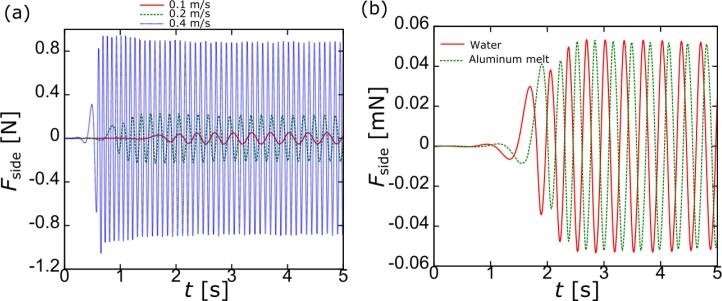
Time variation of force acting on a cylinder parallel to the flow: (a) with different flow velocities in water and (b) with different fluids.

An example of numerically obtained results on the Karman vortices using a large eddy simulation is presented below. A cylinder was fixed at the central part of a two-dimensional calculation domain as shown in Fig. 1. A steady flow was applied from the right side of the domain. The cylinder diameter was set to 8 mm and the flow velocity was set to 0.1, 0.2, and 0.4 m/s. Water or molten aluminum was used as a fluid in the present calculations. Fig. 2 shows the time variation of velocity distribution around the cylinder at different flow velocity in water. The time interval of each picture is 0.02 s. As the flow velocity is increased, the oscillation frequency of Karman vortices behind the tube becomes higher. The time variation of force acting on the cylinder parallel to the flow is shown in Fig. 3 for different flow velocities in water (a) and different liquids at a constant flow velocity of 0.1 m/s (b). As clearly seen from this figure, the frequency of Karman vortices increases with flow velocity. Besides, the frequency is independent of the liquid type as shown in Fig. 3 (b). Therefore, one can conclude that the Strouhal number is kept constant with different flow velocities and the Strouhal number in water should be the same as that in aluminum melt.

**Fig. 4:**
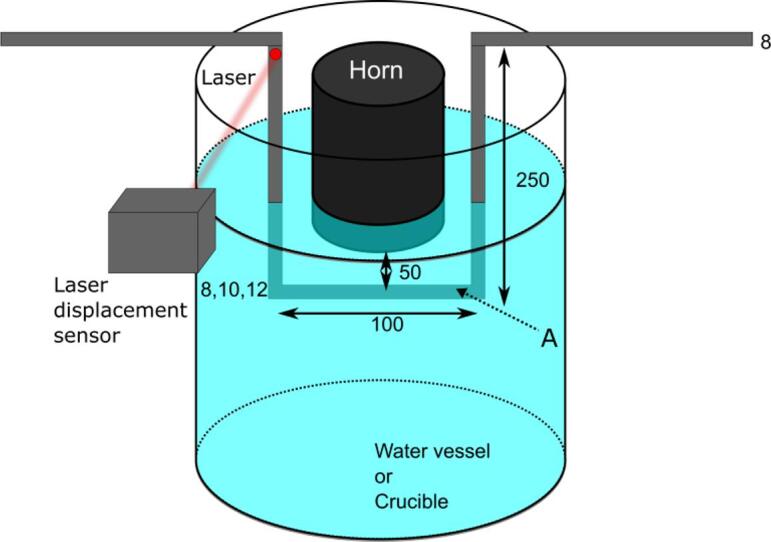
A schematic diagram of experimental setup for frequency measurements.

In the present study the frequency of Karman vortices was determined by measuring the oscillation frequency of a cylinder submerged into liquid. A schematic representation of the experimental setup is shown in Fig. 4. The cylinder was the lower part of a U-shaped stainless steel frame submerged into liquid in such a way that the cylinder was positioned under the sonotrode tip. The acoustic flow induced the cylinder oscillations the frequency of which was measured by a laser displacement sensor, as shown in Fig. 4. The measurement procedure was as follows.

1. Water model experiments were performed to measure the acoustic streaming velocity in water using the particle image velocimetry (PIV) method.
2. The frequency of cylinder oscillations in water was measured under the same conditions by a laser displacement sensor.
3. The Strouhal number was determined from the measurements of acoustic streaming velocity and cylinder oscillation frequency according to Eq. (1).
4. Using the same cylinder and measurement technique, the frequency of cylinder oscillations was measured in aluminum melt.
5. The acoustic streaming velocity in molten aluminum was calculated by inserting the measured frequency into Eq. (1) assuming that the Strouhal number is equal for both fluids, water and aluminum melt.

The experimental details are explained below.

### 2.1 Particle Image Velocimetry (PIV) measurements

**Fig. 5:**
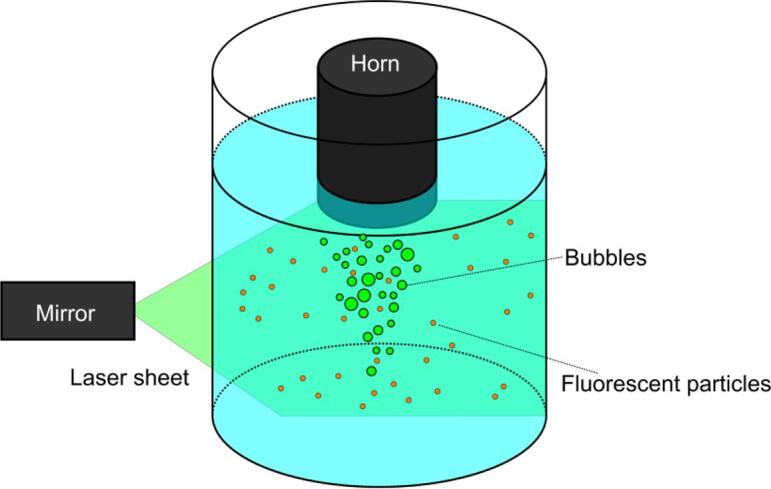
A schematic diagram of experimental setup for PIV measurements.

Fig. 5 shows a schematic drawing of the experimental setup for PIV measurements. The above mentioned ultrasound sonotrode was immerged into a water bath in a circular acrylic container with a diameter of 192 mm and a height of 400 mm. The sonotrode diameter was 48 mm and it was made of silicon nitride. An ultrasonic generator (DG-2000 2 kW, TELSONIC) was used to generate ultrasonic vibrations at a resonant frequency of 20 kHz. The water level in the container was set to 300 mm. Spherical fluorescent particles (FLUOSTAR 0459, Kanomax, diameter 15 μm) in amount of 0.1 g were added to the water bath. The density of particles was 1100 kg/m^3^. An Ar ion laser sheet (GLS3280, Showa Optronics) was irradiated into the central part of container. The laser emitted green beam within a wavelength range 540–550 nm to excite the fluorescent particles. As a result, the particles began to emit orange light at 580 nm. This technique allowed us to separate images of cavitation bubbles and tracer particles by using a cut-off filter passing only the orange light, as shown in Fig. 5. The motion of particles was recorded by a high-speed camera (FASTCAM 1024PCI, Photoron) at a framerate of 500 fps and a shutter speed of 0.001 s. Therefore, only the motion of fluorescent particles could be observed. The recorded particle images were converted into fluid velocity vectors by using a PIV software (FlowPIV, Library).

### 2.2 Frequency measurements by a laser displacement sensor

Fig. 4 shows a schematic diagram of the experimental setup for measurement of cylinder oscillation frequency by a laser displacement sensor. A stainless steel U-shaped frame was immersed into water or aluminum melt. In water measurements, the vessel used was the same as that used for the PIV measurements. In the case of aluminum measurements, pure aluminum (99.9%) was melted in a cylindrical crucible at a temperature of 800 °C using an electrical furnace. The crucible inner diameter and height were 155 and 220 mm, respectively. Similarly to the water case, measurements were carried out by immersing the stainless frame into the aluminum melt. The same ceramic sonotrode was used to irradiate ultrasound waves into molten aluminum. The diameter of U-shaped frame rods was 8 mm except that of part A as shown in Fig. 4. Here cylinders with three different diameters, 8, 10, and 12 mm were used to determine the *St* number. The distance between the cylinder and sonotrode tip was set to 50 mm. The peak-to-peak amplitude (hereafter (p-p) amplitude) of sonotrode tip oscillations was set to 36, 44, 50, 58, and 65 μm, respectively. Although these amplitudes were measured at room temperature in air, the ultrasound generator used has an inbuilt function to maintain the amplitude in a wide range of acoustic load variation. Thus, the amplitude is considered to remain the same in air, water and molten aluminum. The cylinder was forced to oscillate due to Karman vortices generated by the acoustic streaming. The oscillation frequency and amplitude was measured by a laser displacement sensor (LK-G5000, Keyence) at a sampling rate of 2000 Hz. The measurement procedure was as follows

1. Ultrasound was irradiated into the liquid for 10 s to stabilize the acoustic streaming.
2. The cylinder oscillations were measured for 25 s.
3. The measurement data were analyzed using the Fourier fast transformation (FFT).

All measurements were carried out three times to determine the average values.

### 2.3 Cavitation intensity measurements

To explain experimental results on the acoustic streaming, there was a need to measure cavitation intensity in molten aluminum. A high-temperature cavitometer was used for this purpose. Its design and main characteristics have reported in our previous paper [1]. Briefly, the cavitometer measures sound pressure and cavitation noise level in five frequency bands, 0.01–0.3, 0.3–0.6, 0.6–1.5, 1.5–5.0 and 5.0–10.0 MHz. The measurement principle assumes that the larger the measurement signal of high frequency harmonics, the higher is the cavitation intensity. The cavitometer probe was submersed in molten aluminum in such a way that its tip was positioned at a distance of 50 mm from the sonotrode tip. The main purpose of these measurements was to check the effect of acoustic shielding.

## 3 Results

### 3.1 PIV measurement in water bath

**Fig. 6:**
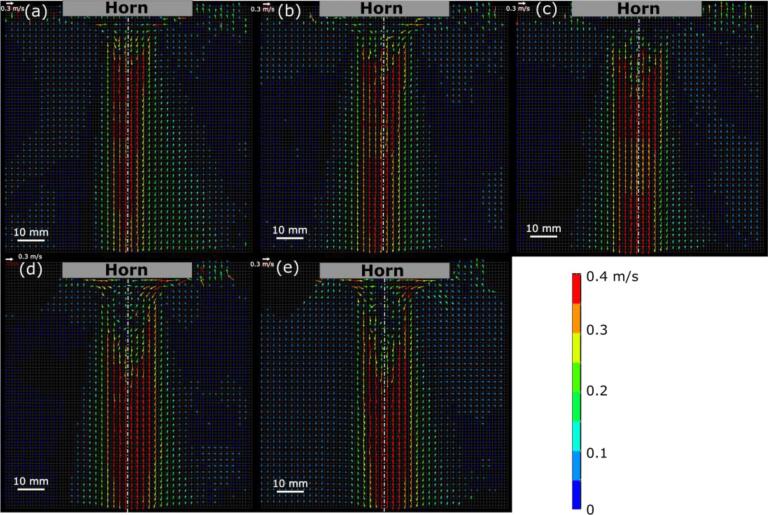
Acoustic streaming velocity measured by the PIV method at different amplitudes, μm (p-p): (a) 36, (b) 44, (c) 50, (d) 58, and (e) 65.

Fig. 6 shows the acoustic flow velocity vectors at different peak-to-peak amplitudes of sonotrode tip oscillations. A strong downward flow is generated, and the flow velocity remains independent of the sonotrode oscillation amplitude. It is seen that the maximum velocity is approximately 0.4 m/s. The flow velocity near the sonotrode tip becomes smaller in all the cases especially at high amplitudes. This phenomenon can be explained by the cavitation bubble cloud. In this measurement, the green light reflected from the bubbles was cut off due to the filter, as mentioned above. Because the volume fraction of cavitation bubbles is increased with the amplitude, the bubbles form a cloud which blocks the passage of beam through this zone. This is why it is difficult to measure the acoustic flow velocity in the immediate vicinity of the sonotrode tip. In the following experiments, the oscillation amplitude was set to 44 μm (p-p).

### 3.2 Karman vortex frequency measurement in water bath

**Fig. 7:**
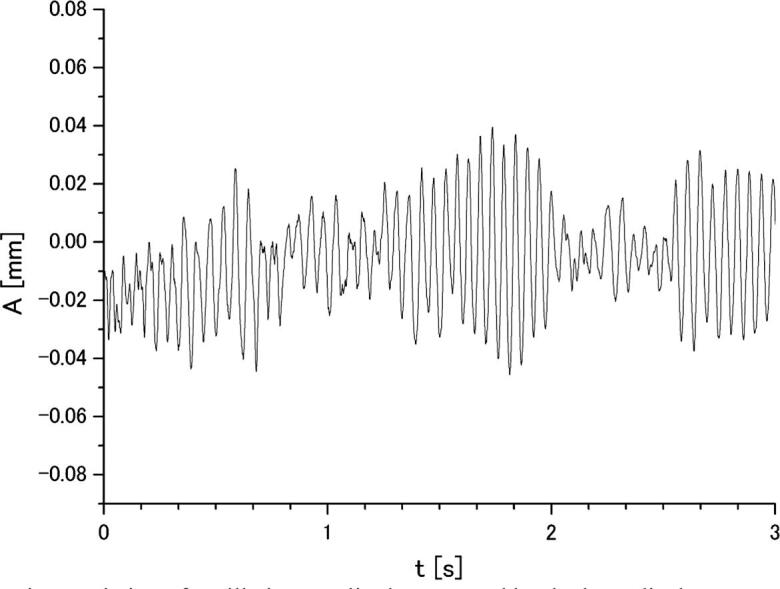
Time variation of oscillation amplitude measured by the laser displacement sensor in water at 44 μm (p-p) amplitude.

**Fig. 8:**
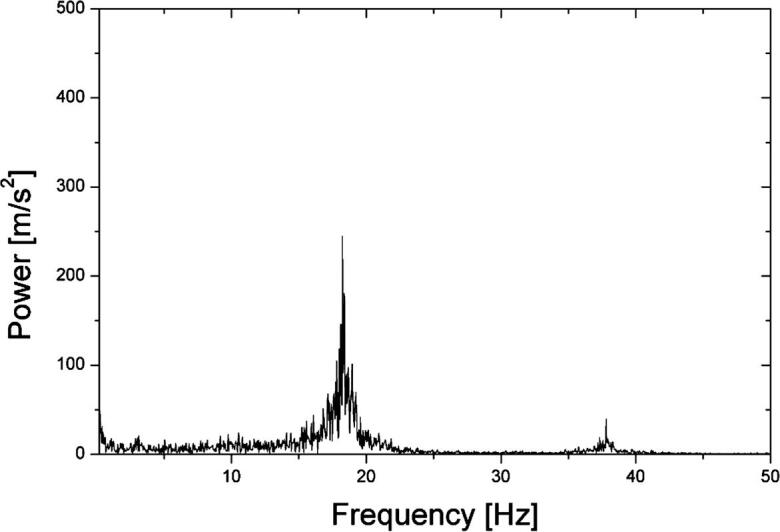
FFT power spectrum for measurement in water at 44 μm (p-p) amplitude.

Fig. 7 shows a typical result on the time variation of cylinder oscillation amplitude in water measured at 44 μm (p-p) amplitude. In this case, the cylinder diameter was 8 mm. The measurement results reveal that the sensor output signal is a superposition of oscillations at different frequencies. Using the FFT analysis, the frequency of the main harmonics was properly determined as shown in Fig. 8.

Thus, the characteristic frequency of cylinder oscillations and corresponding frequency of Karman vorticities is 18.24 Hz, as indicated by the peak in Fig. 8. It is to be noted that the peak is broadening. This is because the acoustic velocity has a non-uniform radial distribution being approximately the same at the central part near the axis and decreasing with the distance from the axis as the distance reaches a certain value. This can be readily seen in Fig. 6. As shown in Fig. 3, the oscillation frequency of Karman vortices is decreased as the fluid velocity becomes smaller. Thus, the above-mentioned decrease of the acoustic velocity with the distance from the central axis could cause shifting the oscillation frequency to lower values that resulted in the peak broadening. The effect of flow non-uniformity on the Karman vortex oscillation frequency has been reported by the other researchers. For example, Chen found that when the velocity in a flow is distributed non-uniformly, the oscillation frequency is deviated by approximately 5% from the uniform (flat) distribution case [22].

Another reason of this broadening is the unstable nature of acoustic streaming. In our experiments, the Reynolds number varied in a range of 3000–5000 that corresponds to a transient flow condition. In other words, the flow becomes unstable that could cause generation of oscillation modes, the frequency of which is higher than the peak one. Certainly, both these reasons could contribute to the increase in the measurement error.

**Fig. 9:**
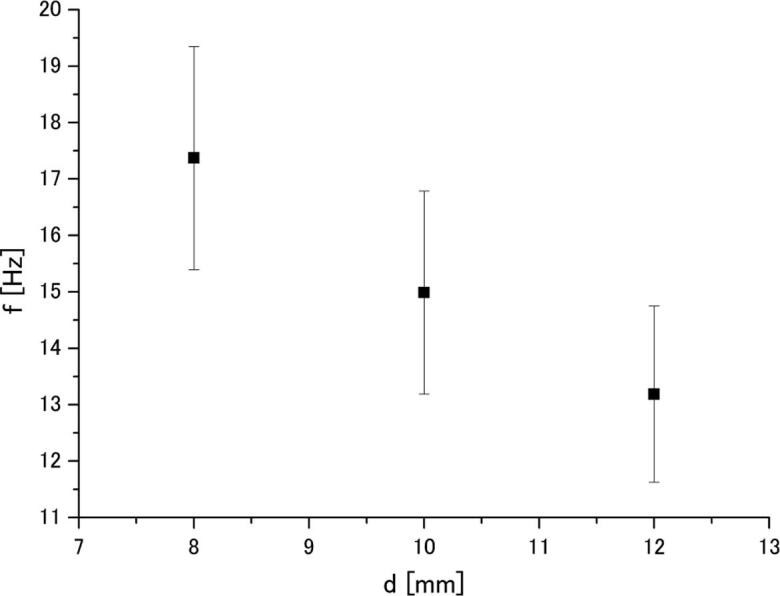
Relationship between frequency of Karman vortices and cylinder diameter in water at 44 μm (p-p) amplitude.

**Fig. 10:**
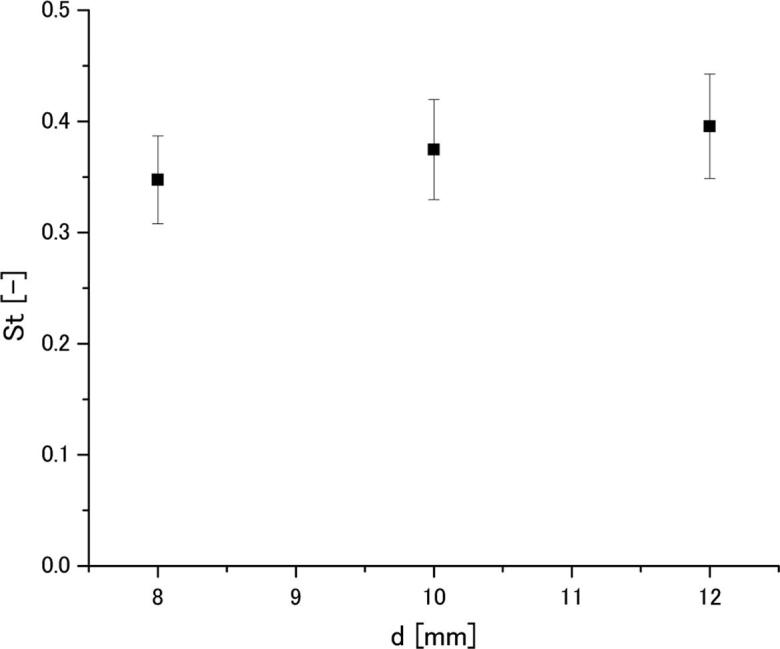
Relationship between *St* number and cylinder diameter in water at 44 μm (p-p) amplitude.

Fig. 9 presents the relationship between frequency of Karman vortices, *f* and cylinder diameter, *d* at 44 μm (p-p) amplitude. It is seen that the frequency of Karman vortices is decreased as the cylinder diameter becomes larger. By substituting these values into Eq. (1), one can obtain the *St* number for each cylinder diameter. The relationship between the *St* number and cylinder diameter at 44 μm amplitude is shown in Fig. 10. Although a tendency for *St* number to be increased with the cylinder diameter is seen in this figure, this increase was found to be statistically insignificant due to the large scattering of measured results. The maximum standard deviation was found to be approximately 10%. Therefore, we considered that the St number does not depend on the diameter under the present experimental conditions and its averaged value is 0.37. This suggests that the Karman vortex frequency can be represented by the *St* number. Based on the above consideration, it is assumed that the same value of *St* number can be used for aluminum melt.

### 3.3 Frequency measurement in molten aluminum

**Fig. 11:**
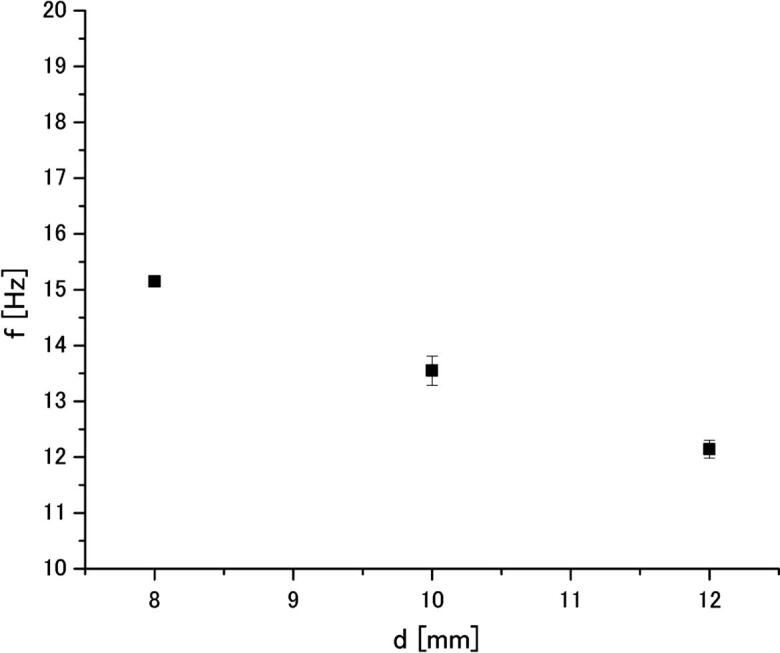
Relationship between the Karman vortex frequency and cylinder diameter in aluminum melt at 44 μm (p-p) amplitude.

**Fig. 12:**
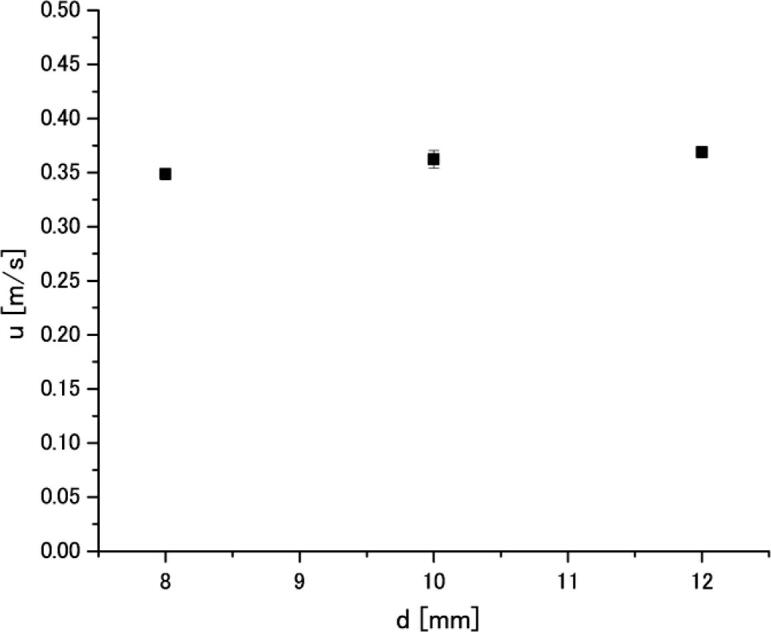
Acoustic streaming velocity in aluminum melt measured at 44 μm (p-p) amplitude.

The frequency of cylinder oscillations was measured in aluminum melt in the same way as that in water. The relationship between the Karman vortex frequency and cylinder diameter is shown in Fig. 11. Similar to the water experiments, the oscillation frequency is decreased with increase of cylinder diameter. Finally, the acoustic streaming velocity was calculated by Eq. (1). The results are shown in Fig. 12. It is seen that the acoustic streaming velocity is also independent of cylinder diameter. This suggests that the velocity measurement using the Karman vortex frequency is valid. The results reveal that the acoustic streaming velocity in aluminum melt is approximately 0.36 m/s, which is by 10 percent less than that in water. The results reveal that the acoustic streaming velocities in aluminum melt and water are 0.36 and 0.4 m/s, which is the same within the measurement error. Therefore, one can conclude that the velocity of acoustic streaming in aluminum melt and water is statistically indistinguishable under the present experimental conditions.

**Fig. 13:**
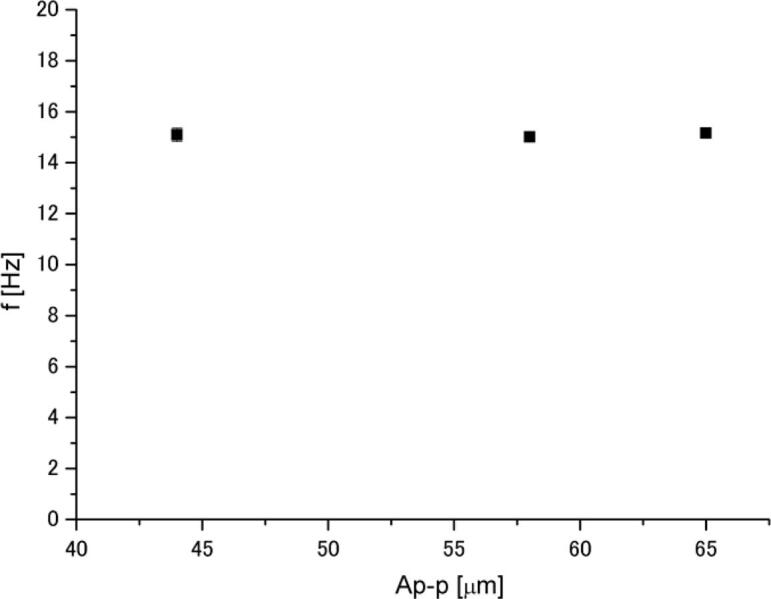
Relationship between frequency of cylinder oscillation and amplitude when the 8 mm cylinder was used.

In addition, the effect of sonotrode vibration amplitude on acoustic streaming velocity in aluminum melt was also investigated. Fig. 13 shows the relationship between the cylinder oscillation frequency and vibration amplitude when the 8 mm cylinder was used. It is readily seen that similar to the water measurements, the frequency is independent of the amplitude. This reveals that the acoustic streaming velocity in molten aluminum remains constant regardless of amplitude.

## 4 Discussion

The above results reveal that the velocity of acoustic streaming in aluminum melt is approximately the same as that in water under given experimental conditions. This is assumed to be caused by a combined effect of attenuation of ultrasound propagation, volume fraction of cavitation bubbles and density of aluminum. As reported in our previous study [7] and by other researchers [4], the attenuation of ultrasound propagation is stronger in aluminum melt compared to that in water. The strong attenuation of ultrasound waves causes the following two phenomena: 1. steep ultrasound pressure gradient just below the sonotrode tip, 2. reduction of cavitation zone in size. The former phenomenon makes the acoustic streaming stronger, while the latter one weakens it. Also, the size of cavitation bubbles is different in water and aluminum melt [9]. Our theoretical analysis indicated that the larger cavitation bubble in aluminum causes higher attenuation of ultrasound waves during their propagation [7]. The volume fraction of cavitation bubble is another factor affecting the velocity of acoustic streaming. However, reliable data on the volume fraction of cavitation bubbles in aluminum melt are unavailable. Finally, the liquid density is another factor affecting the velocity of acoustic streaming. The density of aluminum melt is approximately 2.4 times higher than that of water. When the volumetric force driving the acoustic streaming due to sound pressure attenuation and bubble movement is the same in water and in aluminum melt, the acoustic streaming velocity becomes smaller due to larger density of aluminum melt. This has been confirmed by an earlier study showing that the velocity of acoustic streaming is inversely proportional to the liquid density [17]. As a result of the above factors, the velocity of acoustic streaming in aluminum melt becomes comparable with that in water when the conditions of ultrasound irradiation are kept the same.

We also compared the velocity of acoustic streaming in water with other studies. The acoustic streaming velocity with a large-amplitude oscillation of sonotrode is from 0.3 − 2 m/s depending on the examined conditions [23], [24], [25], [26]. The acoustic streaming velocity in our present experiment was approximately 0.4 m/s, which is in the range of previously reported data as mentioned above. It should be noted that the previous studies carried out a PIV experiment without using fluorescent particles and optical cut-off filter and without eliminating the effect of cavitation bubble movement on the measured flow velocity. The acoustic streaming velocity of the above studies [23], [25] is considered to be overestimated. On the other hand, the measured acoustic streaming velocity is smaller in their study because they used a cut-off filter with fluorescent particles [26]. The acoustic streaming velocity obtained in the present study is close to that in Ref. [26].

Under the present experimental conditions, the acoustic streaming velocity is independent of the amplitude of sonotrode tip oscillations both in water and in aluminum melt. One reasonable explanation of this finding is the effect of cavitation bubble shielding [27]. In our experiments, the amplitude of sonotrode tip oscillations is large enough to cause the cavitation bubble shielding. This shielding effect results in rapid attenuation ultrasound pressure in the immediate vicinity under the sonotrode tip, and then the ultrasound pressure distribution becomes independent of the oscillation amplitude.

**Fig. 14:**
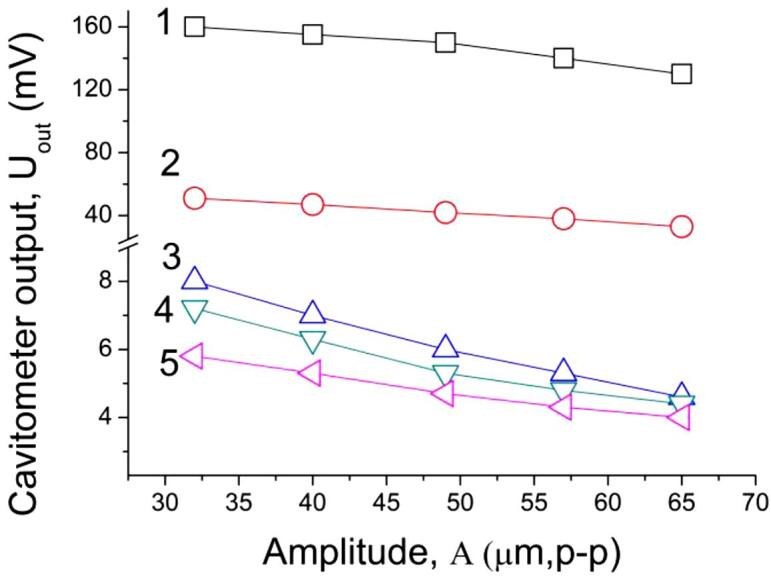
Effect of vibration amplitude on cavitation output voltage in different frequency bands: 1- Total (0.01 ~ 10 MHz), 2–0.3 ~ 0.6 MHz, 3–0.6 ~ 1.5 MHz, 4–1.5 ~ 5.0 MHz, 5–5.0 ~ 10.0 MHz.

To prove the shielding effect in aluminum melt, the cavitation intensity was measured at a location of 60 mm from the sonotrode tip at various amplitudes of its oscillations. Fig. 14 shows the cavitometer output signal, *U*_out_ as a function of sonotrode tip amplitude for different frequency bands of cavitation noise. It is seen that values of *U*_out_ are decreased with the vibration amplitude in all frequency bands suggesting reduction in the cavitation intensity as the amplitude increases. This can be explained only by the acoustic shielding effect. It is notable that the shielding effect appears only at relatively large distances from sonotrode tip. For example, in our previous study [1] the intensity of high frequency harmonics was reported to be increased with vibration amplitude when the cavitometer probe tip was positioned at a distance of 15 ~ 20 mm the sonotrode tip. Under this condition, it is assumed the cavitometer probe tip was inside the high-intense cavitation zone, and no acoustic shielding effect was observed.

## 5 Conclusion

In the present study, the velocity of acoustic streaming in water and molten aluminum was measured based on the measurement of oscillation frequency of the Karman vortices around a cylinder immersed in liquid. In aluminum melt, such measurements were performed for the first time to the best knowledge of the authors. The findings of the present study can be summarized as follows:

1. The velocity of acoustic streaming in water is approximately 0.4 m/s independently of the sonotrode tip vibration amplitude ranged from 36 to 65 μm (p-p). This can be explained by the effect of acoustic shielding under the present experimental condition.
2. The oscillation frequency of Karman vortices depends on the cylinder diameter in such a way that the Strouhal number is kept approximately constant. Under the present experimental conditions, the Strouhal number for Karman vortices is found to be approximately 0.37 in both water and aluminum melt.
3. The velocity of acoustic streaming in aluminum melt was found to be approximately the same as that in water. This is presumably due to different density and different size and number density of cavitation bubbles in water and aluminum.

## CRediT authorship contribution statement

**Takuya Yamamoto:** Writing - original draft, Conceptualization, Methodology, Investigation. **Kazuki Kubo:** Investigation, Formal analysis. **Sergey V. Komarov:** Supervision, Writing - review & editing, Funding acquisition, Project administration.

## Declaration of Competing Interest

The authors declare that they have no known competing financial interests or personal relationships that could have appeared to influence the work reported in this paper.
